# Protocols and assessment procedures in fiberoptic endoscopic evaluation of swallowing: an updated systematic review

**DOI:** 10.1016/j.bjorl.2021.03.002

**Published:** 2021-04-02

**Authors:** Aline Prikladnicki, Márcia Grassi Santana, Maria Cristina Cardoso

**Affiliations:** a Universidade Federal do Rio Grande do Sul, Porto Alegre, RS, Brazil; b Irmandade Santa Casa de Misericórdia de Porto Alegre, Porto Alegre, RS, Brazil; c Universidade Federal das Ciências da Saúde de Porto Alegre, Porto Alegre, RS, Brazil

**Keywords:** Swallowing disorders, Endoscopy, Speech-language and hearing science

## Abstract

**Introduction:**

Neurological alterations can generate swallowing disorders and fiberoptic endoscopic evaluation of swallowing is one of the tests performed for its diagnosis, as well as assistance in dysphagia management.

**Objective:**

To identify and describe a fiberoptic endoscopic evaluation of swallowing standardized protocol for the neurological adult population and its stages.

**Methods:**

Systematic review registered on the PROSPERO platform (CRD42018069428), carried out on the websites: MEDLINE, Cochrane Library and Scielo; published between 2009 and 2020. Randomized clinical trials, cross-sectional, and longitudinal studies were included. Two independent judges evaluated the study design and extracted the data from the selected studies. Doubts regarding inclusion or not of the studies were evaluated by a third judge. Scientific articles included were those with adult neurological remained patients with outcomes: (1) diagnosis of swallowing disorder (2) change in sensitivity in laryngeal region (3) penetration of food offered (4) aspiration of food offered.

**Results:**

3724 articles were initially selected, after personalized search for patients with neurological alterations 101 studies remained. In the end, 21 qualitative studies from 2009 to 2020 remained in the systematic review and they were described in detail and compared. Seven articles used protocols of the institutions in which the research took place and four mentioned using the same protocol. The reliable reproducibility of the protocols is feasible only in three of the articles, even presenting different protocols.

**Conclusion:**

There is no standard or validated protocol to assess the swallowing function of adults with neurological diseases.

## Highlights

• Assessments of neurological populations do not demonstrate standardization in swallowing videoendoscopy.
• Need for standardization of VED protocols for patient diagnosis and management.
• None of the studies used the same protocol.
• The quality of the studies varied widely mainly in their methodologies and protocols.

## Introduction

The fiberoptic endoscopic evaluation of swallowing (FEES) is one of the tests used to assess the function of swallowing and is currently considered a test already established to identify dysphagia in both children and adults. Described in 1988 by Langmore et al.^1^ in a scientific article, in 2001 FEES had its procedures detailed.^2^ A historical study on FEES has recently been published,^3^ describing the evolution of the procedures used to perform the test and its management in dysphagia, as well as some validations in specific populations.

Some published studies on FEES have focused on the validation of protocols for specific populations, such as extubated patients,^4^ head and neck cancer patients,^5^ tracheostomized patients,^6^ vocal fold paralysis patients,^7^ osteopathy,^8^ and myasthenia gravis.^9^ Other studies validated FEES protocols for specific neurological populations, however, not all studies described the protocol used and validated to be used in clinical practice or even to have its study replicated, in order to confirm the results in similar populations.

The focus on the use of FEES in populations of neurological etiology has some particularities, since the examination in the adult neurogenic population may be difficult due to factors such as: the cognitive aspect,^10^, ^11^ trunk and head posture during the exam and the occurrence of fatigue,^12^, ^13^, ^14^ which requires the test interruption and makes the diagnosis and treatment plan difficult.

In general, the FEES assessment protocol is divided into three stages: the first is through careful observation of the anatomy, secretions, and visualization of the movements of the nasal structures when a patient is asked to speak and breathe. Some protocols include assessment of sensitivity in the oropharyngeal region by touching the endoscope in specific regions.^15^, ^16^ The second stage consists of the direct evaluation of swallowing, offering food and liquids in different consistencies. The third stage consists of verifying postural maneuvers, variations in consistencies and observing eating behaviors, directly identifying postures and food consistencies that favor oral intake in a safer way.

Instrumental assessments to evaluate swallowing function have a gold standard in the FEES and in the videofluoroscopic swallowing study (VFSS).^17^

VFSS is a test in which the swallowing process is observed from the time the bolus is captured, passing through all the swallowing phases (oral preparatory phase, oral phase, pharyngeal phase, and esophageal phase).^18^ Therefore, this is an exam that determines the degree (mild, moderate, severe) of swallowing changes. In contrast, the FEES observes the pharyngeal phase of swallowing, however it has the advantage of identifying the exact location of the waste in that phase of swallowing, its quantity, and identifying which best maneuvers performs the partial or total cleaning of this residue. In addition, FEES, due to the fact that it does not use radiation, demonstrates greater ease of reproducibility and replicability, both in inpatients and outpatients. Focusing on the adult neurological population, in its evaluation and therapeutic follow-up, FEES is often indicated.^19^

Therefore, the use of a specific protocol by speech therapists and physicians during FEES performance facilitates the examination and the clinical diagnosis, offering evidence-based recommendations and reducing the rate of variation. The aim of this study is to identify and describe, based on a systematic review of the scientific literature, the FEES protocol to be used in the adult neurological population with details and the possibility of worldwide standardization.

## Methods

A systematic review of the literature was carried out guided by the question: “Is there a protocol for performing FEES and, if so, is it validated to be used universally in patients with neurogenic diseases?”

This study was registered on PROSPERO (CRD42018069428). The databases searched were PubMed/Medline, Cochrane Library, Web of Science and SciELO. The main descriptors related to the investigated theme, crossed, were: FEES evaluation; adults; neurogenic disease; swallowing assessment, as shown in the strategies presented in Table 1. The outcome of each study was considered, that is: presence of a diagnosis of swallowing disorder; impaired laryngeal sensation (at the level of vocal folds); presence of laryngeal penetration of bolus; and/or occurrence of tracheal aspiration.

**Table 1:** Search strategies for the selected databases.

Databases	Search period	DeCS and MesHS descriptors used	Initial result (n)
PubMed/Medline	March to July/ 2018	*Endoscopic swallowing assessment AND assessment procedures fiberoptic endoscopic evaluation of swallowing AND protocols of assessments*	445
	March/2020	*Endoscopic swallowing assessment AND assessment procedures fiberoptic endoscopic evaluation of swallowing AND protocols of assessments*	1546
	March/2020	*Endoscopic swallowing assessment AND assessment procedures AND neurology*	77
Cochrane Library	March/2018	*Endoscopic swallowing assessment AND assessment procedures fiberoptic endoscopic evaluation of swallowing AND protocols of assessments*	83
	March/2020	*Endoscopic swallowing assessment AND assessment procedures fiberoptic endoscopic evaluation of swallowing AND protocols of assessments*	1630
	March/2020	*Endoscopic swallowing assessment AND assessment procedures AND neurology*	14
SciELO	March/2018	*Endoscopic swallowing assessment AND assessment procedures fiberoptic endoscopic evaluation of swallowing AND protocols of assessments*	20
	March/2020	*Endoscopic swallowing assessment AND assessment procedures fiberoptic endoscopic evaluation of swallowing AND protocols of assessments*	20
	March/2020	Endoscopic swallowing assessment AND assessment procedures AND neurology	10

The review included cross-sectional studies, randomized clinical trials, and longitudinal cohort studies, which used FEES as a standard assessment instrument with neurogenic disease patients. Other types of studies or formats were excluded, as well as cross-sectional studies that included children and/or adolescents. The selection of articles covered the period between 2009 and 2019. The survey of bibliographic data took place between March 2018 and March 2020, based on the aforementioned inclusion criteria (Table 1).

The first phase of article selection was the exclusion of duplicate studies, followed by the reading and analysis of titles and abstracts of all identified works. Afterwards there was a complete reading of the selected studies, which led to the exclusion of works that did not meet the review criteria. The selected articles were submitted to methodological evaluation, according to the checklist provided by the report Strengthening the Reporting of Observational Studies in Epidemiology (STROBE)^20^ for cross-sectional studies, which received a score of 1 when the item was considered, of 0 when not contemplated, and of 0.5 when partially contemplated. Afterwards, the averages between the scores assigned by the two evaluators were established. All phases were carried out by two of the authors/researchers, independently. Faced with doubts about whether or not to include the study, the third author/evaluator was called. This study included only articles with at least 70% of the score determined by the STROBE checklist. The arithmetic mean of study scores was 17.86, making up a proportion of 81% of the STROBE score. The included articles were analyzed regarding the possibility of bias, study limitations, number of participants, gender, age, and statistical method (Table 2). All review procedures presented here were conducted in accordance with Checklist Preferred Reporting Items for Systematic Reviews and Meta-Analyzes (Prisma).

**Table 2:** Average among blind evaluators of the published observational studies according to the Strobe tool.

Items/Articles — mean	1	2	3	4	5	6	7	8	9	10	11	12	13	14	15	16	17	18	19	20	21
1. Title and abstract	1	1	1	1	0,75	1	0,75	1	1	1	0,75	1	1	1	1	1	1	1	1	1	1
2. Introduction: context//fundamentals	1	1	1	1	1	1	1	1	1	1	1	1	1	1	1	1	1	1	1	1	1
3. Objectives	0.75	1	0.75	1	1	1	1	0.75	1	0.5	0.75	1	1	1	1	1	1	1	1	1	1
4. Methods: study design	0.5	1	1	0	0.5	1	1	0.5	1	1	0.5	1	1	0.5	0.5	0.5	1	1	1	0.5	1
5. Context	0.5	0.75	1	0.75	0.75	1	0.75	1	1	1	0.75	1	0.5	0.75	1	1	1	1	1	0.75	0.75
6. Participants	1	1	0.5	1	1	1	1	1	1	1	1	1	0.75	0.75	1	1	1	1	1	1	1
7. Variables	1	1	1	1	1	1	1	1	1	1	1	1	1	1	1	1	1	1	1	0.75	1
8. Data sources//measurements	1	1	1	1	1	1	1	1	1	1	1	1	1	1	1	1	0.5	1	1	0.75	1
9. Biases	0.5	0.5	0.25	0.5	0.25	0.5	0.5	0.5	0.25	0.25	0.5	0.25	0.25	0.5	0	0.25	0	0	0.5	0.25	0
10. Sample size	0.5	0.5	0	0	0	1	0.25	0.25	0.25	0.5	0.25	0.5	0	0.25	0.5	0.25	0.5	0	0.5	0.5	0.5
11. Quantitative variables	0.5	0.5	1	1	0.5	1	1	0.5	0.5	0.5	0.5	0.5	0.75	0.5	0.5	0.5	0	0.5	1	0.5	0.5
12. Statistical methods	1	1	0.25	1	1	1	1	1	1	1	1	1	1	1	0.5	1	0	1	1	1	0.5
13. Results: participants	1	1	1	1	0.75	1	0.75	1	1	1	0.75	1	0.75	1	1	1	1	0.5	1	1	0
14. Descriptive data	0.5	0.75	1	0.75	0.75	1	0.75	0.75	1	0.75	0.75	0.75	0.75	0.75	0.75	0.75	0.75	0.5	0.75	0.75	0.25
15. Variable data	1	1	1	1	1	1	1	1	1	1	1	1	1	1	1	1	1	1	1	0.75	1
16. Main results	1	0.75	1	1	1	1	0.75	1	1	1	1	1	1	1	1	1	1	0.75	1	1	0.5
17. Other analyzes	1	1	0.25	1	1	1	0.5	0.25	1	1	0.5	0.5	1	0.5	0.5	1	0	0.5	0.5	0.5	0
18. Discussion: key results	1	1	1	1	1	0.75	1	1	1	1	1	0.75	1	1	0.75	1	1	1	1	1	1
19. Limitations	0.75	0.75	0	1	1	1	1	0	0.25	0.5	1	1	1	1	1	1	1	1	1	0.25	0.75
20. Interpretation	1	1	1	1	1	0.5	1	0.75	1	1	1	1	0.75	1	1	1	1	1	1	1	1
21. Generalization	0.75	1	1	1	1	1	1	1	1	1	1	1	0.25	1	1	1	1	1	1	1	0.5
22. Other information: financing	1	0.5	0	0	1	0.5	1	1	0.5	0	1	1	0	0	0.25	0	0.5	1	0.5	0	1
Total	18.25	19	16	18	18.25	20.25	19	17.25	18.75	18	18	19.25	16.75	17.5	17.25	18.25	16.25	17.75	19.75	16.25	15.25

1. Warnecke et al.^26^; 2. Mandysova et al.^21^; 3. D’ottaviano et al.^31^; 4. Pilz et al.^36^; 5. Somasundaram et al.^37^; 6. Leder et al.^22^; 7. de Lima Alvarenga, et al.^23^; 8. Marian et al.^32^; 9. Nienstedt et al.^27^; 10. Pflug et al.^28^; 11. Umay et al.^40^; 12. Braun et al.^24^; 13. Farneti et al.^41^; 14. Imaizumi et al.^34^; 15. Schröder et al.^35^; 16. Shapira-Galitz et al.^30^; 17. Souza et al.^38^; 18. Souza et al.^39^; 19. Suntrup-Krueger et al.^25^; 20. Warnecke et al.^29^; 21. Gozzer et al.^33^

The arithmetic mean among the assessed works was 17.03 points, with a minimum score of 15.25 and a maximum of 20.25. The minimum score for evaluating the studies was 69% and the average applicability of the studies was 78%.

## Results

The selection of studies carried out by 2 independent evaluators in three databases, as described in the methodology, found a total of 3724 published studies based on the crossed descriptors “fiberoptic endoscopic evaluation of swallowing AND protocols of assessments” and “endoscopic swallowing assessment AND assessment procedures” .

Based on the personalized search and the inclusion of the descriptor “neurology”, aiming at studies performed with adult neurological population, 77 articles were selected on the PubMed platform, 14 in the Cochrane Library, 10 in Scielo, making a total of 101 articles. In search of article eligibility and from reading abstracts, articles that did not meet the inclusion criteria of the study were excluded, 28 articles remaining for full reading, which were analyzed by two blinded evaluators.

**Figure 1:**
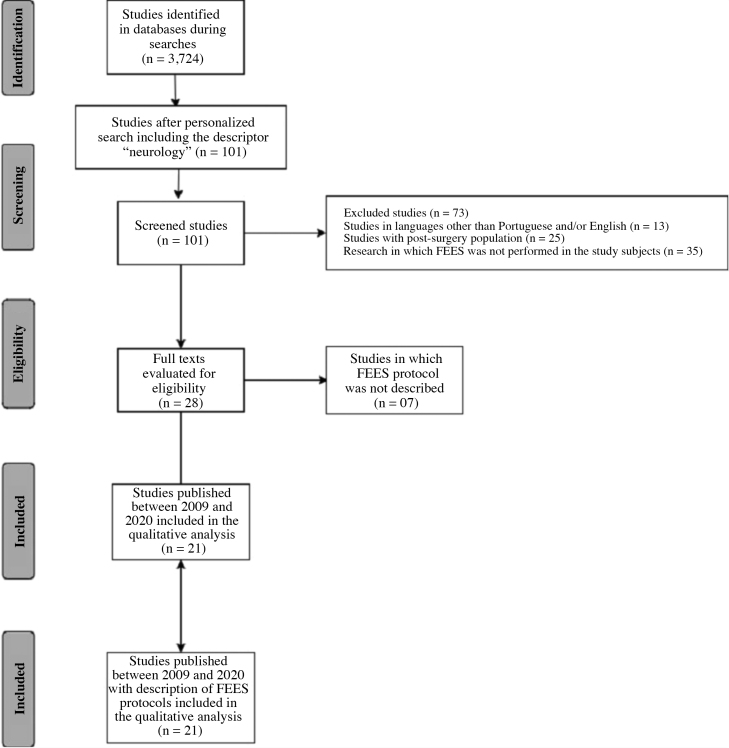
Study search diagram according to Preferred Reporting Items for Systematic Reviews and Meta-Analyzes statement (PRISMA).

Finally, 21 studies published between 2009 and 2020, considered as updated publications, were selected because they were the only ones with complete FEES protocols described in the publications. The description of the search for the articles is shown in Fig. 1 based on PRISMA guidelines (Fig. 1).

Of these, 18 studies are cross-sectional, 02 cross-sectional longitudinal and 01 application of a protocol developed through a cohort. The neurological diseases assessed in the selected studies were: progressive supranuclear palsy (PSP), amyotrophic lateral sclerosis (ALS), elderly patients hospitalized with some health condition, Parkinson’s disease (PD), stroke, myotonic dystrophy type 1 (DM1), myasthenia gravis (MG), elderly >60 years, traumatic brain injury (TBI), and vascular dementia. The description of the articles and the methodological characteristics of the articles are shown in Table 3.

**Table 3:** Analysis of selected studies.

Author, publication date	Neurogenic disease	Number of patients/sex	Associated Evaluation	FEES steps	Tested consistencies (food/utensil)	Volumes	FEES results in swallowing function
Warnecke et al., 2009 ^29^	Acute stroke	153 (80 women)	Dzeiwas protocol performed by neurologist and speech therapist	1. Structural evaluation initially with endoscopy	a. Pasty	a. Teaspoon of puree	6-point scale to determine the severity of dysphagia, where 1 = no laryngeal penetration or laryngotracheal aspiration with soft solid (no change) and 6 = penetration or aspiration with saliva (severe)
					b. Liquid		
					c. Soft-solid	b. Teaspoon of water with food contrast	
			FEES 24 h after hospital admission	2. Evaluation of secretion management			
				3. Functional evaluation of swallowing		c. Small piece of white bread	
						Note: Quantity and number of offers not specified	
Warnecke et al., 2010 ^26^	PSP	18/11 men	Levodopa-test; FEES with monitoring by ENT doctor and speech therapist	1. FEES in the “off” state of levodopa;	a. Pudding (gelatin)	a. 3 × 8 mL pudding	Posteriorleakage of food or liquid;
	PD	15/11 men			b. Liquid (water)	b. 3 × 5 mL liquid	
				2. 200 mg dose of levodopa administered	c. Soft solid (white bread)	c. 3× pieces of bread (3 cm/3 cm/0.5 cm)	Penetration and/or aspiration events;
				3. New FEES was performed after 60 min	Note: All foods colored blue or green		Presence or absence of waste
Mandysova et al., 2011 ^21^	Stroke	87/ND	*Brief Bedside Dysphagia Screening Test*	1. Physical assessment – motor function of muscles and reflexes involved in swallowing;	a. Thick liquid	a. Four teaspoons	Penetration-aspiration scale by Rosenbek et al.
	MG						
	PD						
	ALS		FEES with ENT doctor and nurse monitoring		b. Thin liquid (spoon)	b. Four teaspoons	
	ENT						
				2. Functional assessment of swallowing	c. Thin liquid (glass)	c. 60 mL in the glass	
					d. Assessment of patient voice after swallowing	Note: If the patient coughs, chokes, has a wet voice or leaks from the mouth in <1 min, the test was interrupted.	
					Note: Not specified if the food was colored during the exams		
D’Ottaviano et al., 2013^31^	ALS	11 (6 men)	Protocol described in the study, monitored by ENT doctor and speech therapist	1. Swallowing self-assessment questionnaire	a. Pasty (water plus two tablespoons of the thickener Resource Thicken Up - Nestlé®	a. 5 and 10 mL	Posteriorleakage
						b. 5 and 10 mL	Food residue
				2. Assessment of tongue mobility and fasciculations		c. Half of salt and water cracker	Laryngeal penetration
							Tracheal aspiration
				3. Functional assessment of swallowing	b. Liquid (water)		Timing until tracheal aspiration occurs
					c. Solid (cracker)		
					Note: All foods colored blue.		Response to tracheal aspiration
Pilz et al., 2014^36^	DM1 Controls	45 DM1 (28 men)	Langmore Protocol^2^ Functional Oral Intake Scale (FOIS) Monitored by ENT doctor and speech therapist.	1. Seated patient	a. Thin liquid (water)	a. 10 mL (3 offers)	FOIS scale and visual perception of variables during FEES:
		10 controls (7 women)		2. Evaluation of functionality and morphology of oropharyngeal structures	b. Thick liquid (applesauce)	b. 10 mL (3 offers)	
					c. Solid (cracker)	c. 1 piece of solid	Multiple swallows
					Note: All foods colored blue.		Latency at the beginning of the pharyngeal reflex
				3. Food bolus or liquid inserted into the oral cavity using a syringe			Valecule residue after swallowing
							Residue on piriform sinuses after swallowing
							Laryngeal penetration or tracheal aspiration
Somasundaram et al., 2014^37^	Middle cerebral artery acute stroke	67 (all men)	Initial visit – complete medical history;	FEES performed by an experienced speech therapist and neurologist; Langmore Protocol ^2^;	1. Thickened liquid	a. 3× thickened water	Assessment of dysarthria, dysphonia, volitional cough, and gag reflex;
					2. Semi-solid	b. 3× pudding	
			Physical examination; EAT-10 before FEES;		3. Liquid	c. 3× water	
				1. Structural evaluation	4. Solid	d. 3× white bread	Penetration-aspiration scale
			Cynical Assessment of Swallowing – local protocol; FEES;	2. Observation of secretion or saliva accumulation	Note: All consistencies were stained with blue food coloring.	Note: Quantities not specified.	In the presence of pharyngeal residue, the patient was observed for 2 min to identify voluntary swallowing afterwards, for oral cleaning;
			Note: stroke unit patients screened by doctors and evaluated by a speech therapist 24 h after admission.	3. Functional assessment of swallowing			
Leder et al., 2016^22^	Hospitalized elderly	961 (524 men)	Yale Swallow Protocol FEES – with modified Langmore Standard Protocol, as a complementary assessment for some patients; Monitored by ENT doctor and speech therapist	1. Visualization of the most patent nostril for passing an endoscope without anesthesia;	a. Pasty (pudding)	5 to 10 mL for each consistency	Presence or absence of tracheal aspiration;
					b. Liquid (skim milk)		
					c. Solid (cracker)		
				2. Morphological evaluation of oropharyngeal structures;	Note: Not specified if food was colored during the exams		Functional swallowing defined with absence of aspiration;
				3. Functional assessment of swallowing			Non-functional swallowing with the presence of aspiration in any of the consistencies tested during FEES.
Marian et al., 2017^32^	Stroke	50 (25 each sex)	Screening for swallowing with water, in the presence of predictive symptoms of dysphagia, referral to FEES; Langmore Protocol with modifications; 6-point scale for stroke; Clinical monitoring by neurologist and speech therapist.	1. Patients evaluated in bed with elevated headboard in a stroke unit	a. Pasty (pudding)	3 × 3 mL for each consistency	Penetration-aspiration Scale – FEEDS scale – 6-point dysphagia severity scale in endoscopic evaluation (1 = the best performance and 6 = the worst performance)
					b. Liquid (not specified)		
					c. Soft solid (white bread)		
				2. Endoscope was passed through the most patent nostril with application of local anesthetic	Note: All foods colored blue		
				3. Secretion accumulation in the oropharyngeal region evaluated according to the severity scale			
				4. Functional assessment of swallowing			
de Lima Alvarenga et al., 2018^23^	Elderly >60 years	100 elderly (58 women)	Initial interview Modified Langmore Protocol Medical monitoring.	Self-administered by the participant:	a. Strawberry pudding	a. 10 mL	Evaluated as outcomes:
				1. Assessment of swallowing function	b. Skim milk	b. 50 mL in a glass	1. Saliva stasis in the pharynx
					c. Cracker	c. 1 cracker	2. Pharyngeal residue
					Note: Foods colored green with food coloring.		3. Laryngeal penetration
							4. Laryngotracheal aspiration
							5. Laryngeal sensitivity.
Nienstedt et al., 2018^27^	PD	119 PD	FEES with ENT doctor monitoring;	1. Lidocaine application	a. Liquid	a. 90 mL water	Penetration-aspiration scale, Murray scale short version
		32 Control			b. Solid		
			Assessments: MDS-UPDRS; H&Y scale; NMS-Quest; MOCA DSFS	2. Functional assessment of swallowing	c. Soft solid	b. Cracker (91 mm and 20 g)	
						c. Half a bread with butter (94 × 90 × 9 mm, 28 g)	
Pflug et al., 2018^28^	PD	119 PD	FEES with ENT doctors blinded to disease stages; MDS-UPDRS Evaluation H&Y scale NMS-Quest MOCA Assessment of depression – Beck questionnaire, German version	1. Initial evaluation by ENT doctor with a request to: cough or throat clearing after eating or drinking; history of aspiration or pneumonia;	a. Liquid	a. Teaspoon for water	Scale of swallowing restrictions – SSR
		32 Control			b. Solid		
					c. Soft solid		
						b. 90 mL water with straw	Penetration-aspiration scale
						c. 1 cracker (91 mm, 20 g)	
				2. Functional assessment of swallowing		d. Half a piece of bread with butter (95 × 90 × 9, 28 g)	Premature leakage and waste.
Umay et al., 2018^40^	MG	36 MG (20 women)	FEES	1. Without anesthesia	a. Liquid	a. Water (90 mL)	A score of 1−6 was used for the degree of dysphagia (1 = normal swallowing and 2–6 = dysphagia – from mild to severe.
			Manometry	2. Dzeiwas protocol	b. Semi-solid	b. Yogurt	
		25 Control (14 women)	EAT-10		c. Solid	c. Cracker	
			Surface electromyography				
			VFD				
Braun et al., 2019^24^	Post-stroke elderly	152 (94 men)	GUSS	1. Nasal decongestant application (Xylometazoline) and local anesthesia (2% lidocaine gel)	a. Pasty	a. 3× water with thickener	Rosenbek penetration-aspiration scale.
			FEES considering Langmore standard protocol for signs and symptoms of dysphagia		b. Liquid	b. 3× thin water	
					c. Solid	c. 3× solid (unspecified)	
							Outcomes: FOIS, FEDSS
				2. Observation of anatomical structures, mobility of structures and saliva management		Note: Offer in teaspoon; soup spoon; and sip from glass.	
				3. Functional assessment of swallowing			
Farneti et al., 2019^41^	Different etiologies: PD, vascular dementia, stroke, TBI.	16 adults (11 men)	Own protocol with consistencies based on the global initiative FEES associated with penetration-aspiration scale, FOIS, and DOSS	1. Functional assessment of swallowing	a. Pasty	a. 5cc puree	Videos evaluated by 2 independent and experienced FEES evaluators.
					b. Solid	b. 1∕4 cracker (salt and water)	
					c. Liquid		
						c. 5cc liquid	
							Swallowing performance assessed using: Penetration-aspiration scale, FOIS, and DOSS.
							Outcome: average time for cleaning residues / consistency.
Imaizumi et al., 2019^34^	Elderly people with different comorbidities: cerebrovascular disease, dementia, PD	106 (76 women):	FEES performed on patients at risk for dysphagia based on responses to two questionnaires such as EAT-10 Screening with FEES	1. FEES performed by ENT doctor	a. Degree of saliva accumulation in the vallecula and piriform sinuses;	Saliva	FEES associated with the Penetration-Aspiration Scale
		G1 – detectable swallowing alteration;		2. Food-free assessment based on a system developed by Hyodo et al.	b. Glottic closure reflex with touch of endoscope in epiglottis or arytenoid		Level of care required
			Without food – to identify the severity of swallowing changes				Consciousness level
		G2 – swallowing change not detectable					Ability to eat orally
					c. Reflex of onset of swallowing based on white-out time		Skills in activities of daily living
					d. Pharyngeal cleaning and clearance after swallowing 3 mL of colored water		
Suntrup-Krueger et al., 2019^25^	Acute stroke, recently extubated	133	FEES performed 48 h after extubation monitored by a speech therapist and neurologist.	1- Evaluation of secretion management	a. Pasty	Volumes not specified for each consistency	Sensitivity (intact, reduced, or absent)
					b. Liquid		
					c. Soft solid		
				2- Observation of spontaneous swallowing per minute			
							FEDSS >1 considered as dysphagia
			Extubation Assessments:	3- Assessment of laryngeal sensitivity by touching pharyngolaryngeal structures			3-ounce water swallow test performed 72 h after extubation and 24 h after FEES
			Glasgow coma scale; Body temperature; Heart beats; Systolic pressure; Spontaneous breathing in volume; Positive exhalation pressure; Rapid shallow breathing index				
				4- FEES protocol validated for post-stroke patients			
Schröder et al., 2019^35^	PD	Cohort of 105 patients, 20 selected patients:	Langmore Protocol	1. Functional assessment of swallowing	a. Pasty	a. Green jelly	Premature leakage
					b. Liquid		Penetration-aspiration events
					c. Soft solid	b. Blue colored water	
						c. White bread (3 × 3×0.5 cm)	Residues assessed using dysphagia severity scale of 0–3, where 0 = no swallowing changes and 3 = severe dysphagia (penetration-aspiration with 2–3 consistencies).
		G1 – 10 without signs of dysphagia;					
		G2 – 10 with signs of pharyngeal dysphagia					
							Substance P from saliva was collected in G1 and G2
Shapira-Galitz et al., 2019^30^	Stroke	136 (25 from Kaplan Medical Center and 111 from Sheba Medical Center)	Langmore Protocol with minor modifications	1. Small amount of local anesthesia (2% Lidocaine hydrochloride gel)	a. Pasty	a. Applesauce with green dye (with spoon)	Penetration-aspiration scale
	TBI				b. Solid		
	Degenerative neuromuscular diseases				c. Liquid		
						b. Whole meal bread (two pieces with crust and one without crust)	Residues determined as 0 if absent in all consistencies and as 1 for residue presented in each consistency, with a maximum score of 3 if present in the three consistencies
		51 control		2. Functional assessment of swallowing		c. 3% fat milk with green dye (with straw and straight from the glass)	
						Note: 3 offers of each consistency, with approximately 5cc of volume each bolus	
Souza et al., 2019^38^	DM 1	1 (male, 66 years)	Clinical swallowing evaluation	1. FEES by ENT doctor and speech therapist	a. Pasty	a. Peach flavored dietary juice	Laryngeal sensitivity
			FEES performed by doctor.		b. Thickened liquid		Premature oral leakage
				2. Assessment of laryngeal sensitivity	Note: Consistencies according to IDDSI	b. Juice with instant thickener	
							Pharyngeal waste
				3. Functional assessment of swallowing		Note: All consistencies were stained with blue food coloring.	Laryngotracheal penetration and aspiration
						Consistencies offered in 3, 5 and 10 mL using disposable spoons	
Souza et al., 2019^39^	Stroke	G1: 10 (stroke – 8 men);	FEES performed by physician	1. FEES performed without anesthesia	a. Pasty	Note: All consistencies stained with blue food coloring (5 mL offered), without description of the number of offers and which foods for each consistency.	Pharyngeal waste scale based on the YPRSSRS scale
	ALS				b. Thickened liquid		
	PD	G2: 10 (ALS – 5 men);		2. Functional assessment of swallowing with institutional protocol			
		G3: 10 (PD – 5 men)			Note: Consistencies according to IDDSI		
Institutional protocol for functional swallowing assessment	2. Laryngeal sensitivity was assessed by touch with nasofibroscope on the aryepiglottic and arytenoid folds	20 (13 men)	FEES performed by ENT doctor and speech therapist concomitantly	1. Structures observed in motion, initially with emission of the vowel ∕ i ∕	a. Pasty	Note: Without details of the quantity offered in each consistency	Posteriororal leakage;
					b. Thickened liquid		
					c. Liquid		Pharyngeal residue;
					Note: Consistencies according to IDDSI		Laryngeal penetration;
				3. Functional assessment of swallowing			
							Laryngotracheal aspiration

FEES, fiberoptic endoscopic evaluation of swallowing; ENT doctor, otorhinolaryngologist; PSP, progressive supranuclear palsy; PD, Parkinson's disease; MG, myasthenia gravis; ALS, amyotrophic lateral sclerosis; ND, no data; DM 1, Muscular Dystrophy type 1; FOIS, Functional Oral Intake Scale; FEEDS, Functional Evaluation of Eating Difficulties Scale; MDS-UPDRS, Movement Disorder Society’s Unified Parkinson’s Disease Rating Scale; H&Y, Hoehn & Yahr scale; NMS-Quest, Non-Motor Symptoms Assessed by Non-Motor Symptoms Questionnaire; MOCA, Montreal Cognitive Assessment; DSFS, Drooling Severity and Frequency Scale; mL, milliliter; mm, millimeter; mg, milligrams; cc, cubic centimeter; SSR, Sympathetic Skin Responses; VFD, Videofluoroscopy of Deglutition; GUSS, Gugging Swallowing Screening; DOSS, Dysphagia Outcome and Severity Scale; IDDSI, International Dysphagia Diet Standardization Initiative; YPRSSRS, Yale Pharyngeal Residue Severity Rating Scale.

The methodological evaluation of the studies using the STROBE report, by individual evaluation of two blinded and independent evaluators, and the hypothesis for this review found 21 studies which were selected for this systematic review, 5 of them with high score,^21^, ^22^, ^23^, ^24^, ^25^ and one highlighted due to its results and statistical analysis performed.^25^

When verifying the risk of bias within studies, some studies have exposed their limitations and were found in Warnecke et al.^26^ that to avoid the expectation bias, the FEES performed in the study were evaluated randomly by two independent judges; that is, out of the order in which the exams were performed. Alvarenga et al.^23^ reported as one of the limitations of the study a probable sample bias, since the patients who accepted it probably did so due to presenting swallowing symptoms. Braun et al.^24^ reported a possible selection bias when researching patients in the intensive care unit, demonstrating that the study sample was more severely affected. Suntrup-Krueger et al.^25^ pointed out limitations on possible biased results of the study due to the fact that in the intensive care unit there is a high knowledge of post-extubation dysphagia. Nienstedt et al.^27^ and Pflug et al.^28^ stated that they minimized selection bias and Warnecke et al.^29^ commented on a possible selection bias based on the inclusion and exclusion criteria of the study. Shapira-Galitz et al.^30^ suggested a possibility of assessment bias at the time when the researchers needed to read the questionnaire for patients over the phone. D’Ottaviano et al.,^31^ Leder et al.,^22^ Mandysova et al.,^21^ Marian et al.,^32^ Gozzer et al.,^33^ Imaizumi et al.,^34^ Schröder et al.,^35^ Pilz et al.,^36^ Somasundaram et al.,^37^ Souza et al.,^38^ Souza et al.,^39^ Umay et al.,^40^ and Farneti et al.^41^ did not describe the bias assessment.

The number of patients evaluated in the selected studies ranged from 1 to 961, all of them being adults and or elderly, both men and women, and 6 of the studies carried out an evaluation in a control group compared to age matching.^25^, ^27^, ^28^, ^30^^,^^36^, ^40^

Among the protocols presented in the 21 studies, 7 of the articles used protocols from the institutions where the research was carried out or even protocols only detailed in the articles; 2 used the protocol by Dzeiwas et al.^42^; 2 studies used the Langmore protocol^1^; 2 used the Langmore protocol^43^; 4 used the Langmore protocol^2^; 1 article performed FEES evaluations with protocol by Warnecke et al.^9^; 1 study evaluated using the brief bedside dysphagia screening test^21^; 1 used the FEES levedopa-test^26^; and 1 performed evaluations without the use of food using the protocol by Hyodo et al.^44^ However, even though studies have mentioned the use of the same protocol to perform the FEES, in the description of the protocols, we observed differences in the chosen consistencies, foods and volumes, not characterizing the same protocol. The way in which FEES images were captured was not discussed in the articles, which is why it was not discussed in this systematic review.

Detailing the protocols, only 3 of the studies cited the international dysphagia diet standardization initiative (IDDSI)^45^ as a basis for standardizing the food consistencies offered during the FEES of the studies, with thick and liquid consistencies being offered in these studies. One of the studies included the evaluation of liquid in addition to the other consistencies mentioned. Three of the articles presented better and more accurate details of the protocols used to perform FEES, Warnecke et al.,^26^ Shapira-Galitz et al.^30^ and Souza et al.^38^, specifying the tested consistencies, declaring the foods that were used for each consistency, the quantity offered at each moment, and how many times each consistency was offered. Of the 21 studies, 11 mentioned having used food coloring to contrast the color of the food in relation to the structures, blue or green and possible secretions present during the exam.

Among the outcomes assessed by de Lima Alvarenga et al.,^23^ Gozzer et al.,^33^ Souza et al.^38^, and Souza et al.,^39^ there were: posterior oral leakage, pharyngeal residues, laryngeal penetration, laryngotracheal aspiration, and laryngeal sensitivity. The study by Souza et al.^39^ evaluated mainly residues, all of them according to each tested consistency. The studies by Warnecke et al.,^9^ Warnecke et al.,^26^ Marian et al.,^32^ and Braun et al.^24^ used the severity scale for dysphagia in their outcomes, the fiberoptic endoscopic dysphagia severity score (FEDSS).^9^ As for the autonomy and capacity for oral intake based on the Functional Oral Intake Scale (FOIS), they were assessed as outcomes by Leder et al.,^22^ Farneti et al.,^41^ Imaizumi et al.,^34^ and Shapira-Galitz et al.^30^ In the study by Imaizumi et al.,^34^ the International Classification of Functionality was also used for the assessment. The detailed outcomes in each study separately are shown in Table 4.

**Table 4:** Prevalence and swallowing outcomes assessed and demonstrated by the selected studies.

Study authorship, publication date	Swallowing outcomes				Absolute number of patients with the outcome/total of patients (affectation disease)			
Warnecke et al., 2009^9^	FEDSS				Number of patients who presented each score			
	1				73			
	2				25			
	3				20			
	4				15			
	5				12			
	6				8			
	FEDSS prediction for modified ranking scale							
	Independence (mRS 0–2)				76 (49.7%)			
	Dependency (mRS 3–6)				77 (50.3%)			
Warnecke et al., 2010^26^	Dysphagia severity							
	Non-relevant findings				3∕18 (PSP)		2∕15 (PD)	
	Mild dysphagia				7∕18 (PSP)		5∕15 (PD)	
	Moderate dysphagia				5∕18 (PSP)		3∕15 (PD)	
	Severe dysphagia				3∕18 (PSP)		5∕15 (PD)	
Mandysova et al., 2011^21^	Change in FEES × Change in BBDS				31 (87) × 66 (87); S = 87.1%; E = 30.4%			
	Change in FEES × Change in BBDS neurological patients				21 (72) × 57 (72); S = 95.2%; E = 27.5%			
D’Ottaviano et al., 2013^31^	Changes in the swallowing phases							
	Oral preparation				7/11 (ALS)			
	Oral and pharyngeal transit				11/11 (ALS)			
	Pharyngeal phase				11/11 (ALS)			
	Laryngeal penetration or tracheal aspiration				10/11 (ALS)			
Pilz et al., 2014^36^	Aspiration of thin liquid				17/45 (DM1)			
	Aspiration of thick liquid				02/45 (DM1)			
	Mean difference between DM1 × controls							
	Thin liquid				0.56 (0.17. 0.95)			
	Thick liquid				1.27 (0.90. 1.64)			
	Solid				1.63 (0.46. 5.87)			
	The major difference between groups is in relation to a larger piece of solid compared to the liquid.							
Somasundaram et al., 2014^37^	Clinical evaluation outcomes and FEES, n (%)				Dysphagia (n = 41)		Without dysphagia (n = 26)	
	Dysarthria				9 (22)		10 (39)	
	Dysphonia				4 (10)		7 (27)	
	Altered gag reflection				13 (32)		2 (8)	
	Altered voluntary cough				26 (63)		8 (31)	
	Cough after swallowing				25 (61)		5 (19)	
	Vocal alteration after swallowing				1 (3)		1 (4)	
Leder et al., 2016^22^	Patients’ oral intake status							
	Men							
	Oral route				392∕961			
	Nothing by mouth				132∕961			
	Women							
	Via oral				329∕961			
	Nothing by mouth				105∕961			
Marian et al., 2017^32^	Dysphagia severity scale (FEEDS)							
	Grade 1 normal				0/50 (stroke)			
	Grade 2				0/50 (stroke)			
	Grade 3				24/50 (stroke)			
	Grade 4				6/50 (stroke)			
	Grade 5				18/50 (stroke)			
	Grade 6 severe				0/50 (stroke)			
de Lima Alvarenga et al., 2018^23^	Saliva stasis				94 (no) 6 (yes)			
	Pharyngeal residue				61 (no) 39 (yes)			
	Laryngeal penetration				91 (no) 9 (yes)			
	Aspiration				98 (no) 2 (yes)			
	Laryngeal sensitivity				8 (no) 92 (yes)			
Nienstedt et al., 2018^27^	DSFS score	PD patients (119)	PAS 1–2 (80)	PAS 7–8 (28)	PD patients (119)		Controls (32)	
	2 (moist lips only)	59 (50%)	46 (58%)	11 (39%)	88 (74%)		28 (88%)	
	4	18 (15%)	14 (18%)	4 (14%)	25 (21%)		3 (9%)	
	5	20 (17%)	11 (14%)	5 (18%)	3 (3%)		0 (0%)	
	6	10 (8%)	5 (6%)	3 (11%)	3 (3%)		1 (3%)	
	7	8 (7%)	4 (5%)	2 (7%)				
	8	1 (1%)	0 (0%)	1 (4%)				
	9 Sialorrhea (constantly wetting clothes, hands, objects)	3 (3%)	0 (0%)	2 (7%)				
Pflug et al., 2018^28^	Parkinson’s Disease Patients (119)							
	Presence of dysphagia, n (%)				113 (95)			
	Laryngeal penetration or aspiration, n (%)				66 (55)			
	Aspiration alone, n (%)				30 (25)			
	Consistency with higher percentage of aspiration				Liquid (water)			
	PAS of patients with water aspiration, n (%)				7–8. 28 (23.5)			
	PAS of patients with bread-and-butter aspiration, n				7–8. 5 of the previous 28			
	SBP 2–6, n (%)				37 (31%)			
	Waste in general, most commonly with bread, n (%)				111 (93%)			
	Build-up with bread				60 (50%)			
	Build-up considered severe				23 (19%)			
	Premature leakage (score >1) for water, n (%)				11 (8)			
	Premature leakage (score >1) for cracker, n (%)				21 (18)			
	Premature leakage (score >1) for bread, n (%)				4 (3)			
Umay et al., 2018^40^	Outcomes in EAT-10 and FEES				EAT-10		FEES	
	Group 1 (n = 24) without dysphagia				9 (37.5%)		0	
	Group 2 (n = 12) with dysphagia				10 (83.3%)		11 (91.7%)	
	Group 3 (n = 25) healthy controls				2 (8%)		0	
Braun et al., 2019^24^	FEDSS (2–6) determining dysphagia				110 (72.4%)			
	FOIS (diet modification)				105 (69.1%)			
					48 (31.6%) with oral restriction			
					57 (37.5%) decreased restrictions			
					76.6% nothing by mouth (did not change the initial outcome before and after FEES)			
Farneti et al., 2019^41^	Time – Consistency – FOIS scale:							
	Total time – pasty				−1.32			
	Time in sec – pasty				1.12			
	Total time – regular (solid)				−1.92			
	Time in sec – regular				−2.43			
	Total time – liquid				−0.43			
	Time in sec – liquid				−0.90			
	Time-Consistency-DOSS scale:							
	Total time – pasty				−0.29			
	Time in sec – pasty				0.02			
	Total time – regular (solid)				−1.33			
	Time in sec – regular				−0.93			
	Total time – liquid				−0.48			
	Time in sec – liquid				−0.63			
Imaizumi et al., 2019^34^	Outcomes assessed:				Swallowing disorder not detectable (n = 64)		Detectable change in swallowing (n = 42)	
	FEES score				2		5	
	Laryngotracheal aspiration (number of patients)				7		17	
	Mean laryngeal penetration-aspiration score				1		2	
	Ability to eat by mouth (International Classification of Functionality) (number of patients) level 2							
Suntrup-Krueger et al., 2019^25^					Successful extubation (101)		Reintubation (32)	
	The 3-ounce water swallow test							
	Time after extubation, hours				16.6 ± 15.5		18.1 ± 43.2	
	Test failure, n (%)				12 (17.6)		13 (68.4)	
	Secretion assessment, n (%)							
	Normal				70 (82.4)		6 (23.1)	
	Vallecula				7 (8.2)		1 (3.8)	
	Laryngeal vestibule, temporarily				7 (8.2)		7 (26.9)	
	Laryngeal vestibule, permanently				1 (1.2)		12 (46.2)	
	Murray’s Secretion Scale				0.3 ± 0.7		2.0 ± 1.2	
	Frequency of spontaneous swallowing, n (%)							
	0 min				0 (0.0)		8 (30.8)	
	1–3 min				32 (37.6)		15 (57.7)	
	>3 min				53 (62.4)		3 (11.5)	
	Pharyngeal sensitivity, n (%)							
	Intact				40 (47.1)		1 (3.8)	
	Reduced				15 (17.6)		12 (46.2)	
	Absent				4 (4.7)		7 (26.9)	
	Not specified				26 (30.6)		6 (23.1)	
	Pasty consistency, exposed n (%)				73 (85.9)		7 (26.9)	
	Penetration				13 (15.3)		3 (11.5)	
	Aspiration				4 (4.7)		3 (11.5)	
	Liquid, exposed n (%)				77 (90.6)		19 (73.1)	
	Penetration				37 (43.5)		18 (69.2)	
	Aspiration				25 (29.4)		15 (57.7)	
	Soft solid, exposed n (%)				33 (38.8)		0 (0.0)	
	Penetration				1 (1.2)		–	
	Aspiration				0 (0.0)		–	
	Posterior leakage							
	Without leakage				21 (24.7)		0 (0.0)	
	In vallecula				21 (24.7)		2 (7.7)	
	In pyriform sinus				14 (16.5)		4 (15.4)	
	In laryngeal vestibule				17 (20.0)		17 (65.4)	
	Not specified				12 (14.1)		3 (11.5)	
Schröder et al., 2019^35^	Discrete pharyngeal residues, n (%)				10 (50%)			
	Location of these residues, valleculae				10 (100%)			
	Location of these residues, pyriform sinuses				3 (30%)			
	Premature leakage				0 (0%)			
	Penetration/aspiration events				0 (0%)			
	Concentration of substance P in saliva							
	In patients with pharyngeal dysphagia				9.644 pg∕mL			
	In control patients				17.591 pg∕mL			
Shapira-Galitz et al.,2019^30^	Outcomes in patients with neurological diagnoses:				54 (39.7%)			
	EAT-10 Hebrew validation				15.87 ± 8.98			
	Penetration-aspiration scale				4.43 ± 3.04			
	FEES score				2.56 ± 2.0			
	FOIS – Functional Oral Intake Scale				5.85 ± 1.42			
Souza et al., 2019^38^					First FEES		Last FEES	
					5 mL	10 mL	5 mL	10 mL
	Pasty							
	Posterior oral leakage				1	1	1	NT
	Pharyngeal residues in valleculae				2	2	3	NT
	Pharyngeal residues in pyriform sinuses				1	2		NT
	Laryngeal penetration				0	0	3	NT
	Laryngotracheal aspiration				0	0	0	NT
	Thickened liquid				1	1	1	NT
	Posterior oral leakage				1	2	2	NT
	Pharyngeal residues in valleculae				1	2	2	NT
	Pharyngeal residues in pyriform sinuses				1	1	2	NT
	Laryngeal penetration				3	3	5	NT
	Laryngotracheal aspiration				0	00		NT
	Liquid							
	Posterior oral leakage				1	1	1	NT
	Pharyngeal residues in valleculae				1	1	1	NT
	Pharyngeal residues in pyriform sinuses				1	1	1	NT
	Laryngeal penetration				3	3	5	NT
	Laryngotracheal aspiration				0	0	7	
Souza et al., 2019^39^	Total frequency of residues in pasty and liquid consistencies				Presence		Absence	
	Pasty (n = 30)				19 (63.33%)		11 (36.67%)	
	Thickened liquid (n = 27)				16 (59.26%)		11 (40.74%)	
	Residues in valleculae, pasty consistency				Yale scale (0–2)		Yale scale (3–4)	
	GI				9 (90%)		1 (10%)	
	GII				9 (90%)		1 (10%)	
	GIII				9 (90%)		1 (10%)	
	Consistency of residues in pyriform sinuses							
	Pasty							
	GI				10 (100%)		0 (0%)	
	GII				9 (90%)		1 (10%)	
	GIII				10 (100%)		0 (0%)	
	Consistency of residues in valleculae, thickened liquid							
	GI				9 (100%)		0 (0%)	
	GII				8 (89%)		1 (11%)	
	GIII				9 (100%)		0 (0%)	
	Consistency of residues in pyriform sinuses, thickened liquid							
	GI				9 (100%)		0 (0%)	
	GII				8 (89%)		1 (11%)	
	GIII				9 (100%)		0 (0%)	
Gozzer et al., 2019^33^	Outcomes assessed:				Liquid	Thickened liquid	Pasty	
	Posterior oral leakage				10 (55%)	10 (52.6%)	10 (50%)	
	Pharyngeal residue				4 (22.2%)	8 (42.1%)	8 (40%)	
	Laryngeal penetration				7 (38.8%)	5 (26.3%)	6 (30%)	
	Laryngotracheal aspiration				3 (16.6%)	1 (5.2%)	1 (5%)	

BBDS, brief bedside dysphagia screening; S, sensitivity; E, specificity; FEEDS, Functional Evaluation of Eating Difficulties Scale; PSP, progressive supranuclear palsy; PD, Parkinson’s disease; FEES, fiberoptic endoscopic evaluation of swallowing; FEDSS, fiberoptic endoscopic dysphagia severity score; ALS, amyotrophic lateral sclerosis; DM 1, muscular dystrophy type 1; PAS, penetration-aspiration scale; EAT-10, Eating Assessment Tool; FOIS, Functional Oral Intake Scale; NT, not tested.

## Discussion

The objective of this systematic review of identifying a standardized and validated protocol for endoscopic evaluation of swallowing in patients with underlying neurogenic disease has not been achieved. All studies evaluated and selected for this study used described and detailed protocols, but none were validated. Mandysova et al.^21^ developed a dysphagia screening test for bedside application and validated it based on FEES, but it is not a validated FEES protocol. Dziewas et al.^42^ have been mentioned in some of the articles as a validated FEES protocol, however it is the development and validation of a new score for the assessment of dysphagia severity, which does not correspond to a validated FEES protocol for the neurological population. The Hyodo score^44^ was developed and validated to identify the presence and degree of dysphagia, indirectly, that is, without the use of liquids and food, only based on secretion management and intraoral sensitivity in neurological patients, consequently not characterizing a FEES protocol, but an evaluation score. As for all studies that cited the Langmore protocol, in different years of publication (1988, 1998, 2001) and updates, these are considered guidelines, as the author says in a recent 2017 article,^3^ requiring validation for different populations.

Regarding the protocols presented by the selected studies, 7 out of 21 studies used the consistency of pureed, liquid, and soft & bite sized, 6 of the studies used pureed, liquid and solid. The other studies used different consistencies, as an example of liquid and slightly thick only. Considering the existence of consistency standardization based on IDDSI, an international diet standardization initiative^45^ since 2015 and updated in 2019, only Souza et al.,^38^ Souza et al.,^39^ and Gozzer et al.^33^ used international validation. IDDSI favors the use of the same nomenclature worldwide, facilitating the standardization and validation of assessment protocols such as FEES for neurological populations, for example. However, 13 of the 21 studies were published after 2015, the year in which IDDSI was created and published, and did not use international standardization, making it difficult to validate a protocol.

The volumes presented in the study protocols were widely different, varying from studies testing all consistencies in 3, 5 and 10 mL, others testing only 10 mL three times and others identifying volumes as the size of the spoon offered, with the teaspoon being specified, to studies that did not specify volumes or number of offers or even the utensils used.

Some of the utensils used in some studies were straw, glass, but without specifying the size or even diameter of each utensil, making it difficult to understand the quantity offered to the study participant. Therefore, the use of spoons, straws or cups without specifying size and quantity cannot be characterized as a description of volumes, as there are different diameters and sizes for each of these utensils. Additionally, studies citing the same protocol in the description of the article used different consistencies and volumes, consequently not being the same protocol.

Finally, the presence of a speech therapist to provide food and guide the swallowing of patients during FEES alongside the doctor who performs endoscopy was mentioned in 11 of the 21 selected studies. It is clear that the evaluation of swallowing and its possible changes during FEES is of exclusive medical responsibility in Brazil, while the role of the speech therapist is to monitor the evaluation and verify the patient’s responses to body maneuvers for food. Teamwork is the gold standard for an accurate diagnosis and determination of the appropriate therapeutic plan for that patient.

Regarding the quality of the studies, even considering a high cut-off point, most studies were evaluated as good or satisfactory, making it difficult to consider any of these protocols for standardization and/or validation for FEES in the neurological population. However, three of these studies were the ones that best detailed their procedures and protocol for FEES evaluation.^26^, ^30^, ^38^ Three studies brought all the items meticulously detailed since the tested consistencies, one of them using the IDDSI, the volumes offered in units of “mL” or “cc”, mentioned the use of food coloring to favor contrast in relation to the structures and secretions of the organism, food and liquids offered according to each consistency, how many times each one was offered, and which utensils were used to offer each consistency. The way in which FEES images were captured was not discussed in the articles, which is why they are not highlighted in this systematic review.

However, none of these protocols have been validated for the neurological population. The lack of a validated protocol makes it difficult to widely standardize the assessment and changes the therapeutic approach, since the entire rehabilitation is based on a detailed, accurate and reliable assessment. Adequate and correct diagnosis is the basis for any rehabilitation and management of dysphagia in adult patients with neurological disease.

The importance of having a standardized and validated protocol for neurological populations, including specific populations, is crucial because each neurological disease has its own particularities and pathophysiology, as well as the presentation of dysphagia. Dysphagia characteristics vary widely according to the neurological diagnosis. The literature details these differences regarding dysphagia in Parkinson’s disease,^46^ dysphagia in supranuclear progressive paralysis (PSP),^47^ dysphagia in amyotrophic lateral sclerosis (ALS),^48^ as well as dysphagia in traumatic brain injury (TBI),^49^ among others.

Some of the limitations faced in this systematic review were the possible bias in evaluating the quality of the studies, due to the fact that one of the blinded evaluators was the same to account for the averages of the items and the final score and the non-use of the third evaluator in this phase of the study. In addition to the possible bias in the selection of studies, as we have delimited more recent research (from the last eleven years), we may have left out historically important research for this topic, although mentioned in the introduction and discussion.

The lack of a standardized and validated protocol for the adult population with neurogenic diseases significantly limits a detailed, accurate, and focused assessment of the possible swallowing difficulties faced by these patients. The clinical diagnosis of dysphagia may be underestimated or overestimated according to the protocol used and outcomes assessed. This systematic review, the first in the field, highlights the need to validate protocols with a focus on adults with underlying neurogenic diseases considering the characteristics of dysphagia and its pathophysiology. Adequate, reliable and accurate diagnosis is the basis for the management of swallowing in these populations.

## Conclusion

The reliable reproducibility of the protocols is only feasible in three of the articles, even with different protocols, but none were standardized or validated for the adult neurological population.

## Conflicts of interest

The authors declare no conflicts of interest.

## Acknowledgements

This study was financed in part by the Coordenação de Aperfeiçoamento de Pessoal de Nível Superior - Brasil (10.13039/501100002322CAPES) - finance code 001.

## References

[1] Langmore S.E., Schatz K., Olsen N. (1988). Fiberoptic endoscopic examination of swallowing safety: a new procedure. Dysphagia.

[2] Langmore S. (2001).

[3] Langmore S.E. (2017). History of fiberoptic endoscopic evaluation of swallowing for evaluation and management of pharyngeal dysphagia: changes over the years. Dysphagia.

[4] Ajemian M.S., Nirmul G.B., Anderson M.T., Zirlen D.M., Kwasnik E.M. (2001). Routine fiberoptic endoscopic evaluation of swallowing following prolonged intubation: implications for management. Arch Surg.

[5] Deutschmann M.W., McDonough A., Dort J.C., Dort E., Nakoneshny S., Mathews T.W. (2013). Fiber-optic endoscopic evaluation of swallowing (FEES): predictor of swallowing-related complications in the head and neck cancer population. Head Neck.

[6] Leder S.B., Ross D.A. (2010). Confirmation of no causal relationship between tracheotomy and aspiration status: a direct replication study. Dysphagia.

[7] Ollivere B., Duce K., Rowlands G., Harrison P., O’Reilley B.J. (2006). Swallowing dysfunction in patients with unilateral vocal fold paralysis: aetiology and outcomes. J Laryngol Otol.

[8] Seidler T.O., Alvarez J.C.P., Wonneberger K., Hacki T. (2009). Dysphagia caused by ventral osteophytes of the cervical spine: clinical and radiographic findings. Eur Arch Otorhinolaryngol.

[9] Warnecke T., Teismann I., Zimmermann J., Oelenberg S., Ringelstein E.B., Dziewas R. (2008). Fiberoptic endoscopic evaluation of swallowing with simultaneous Tensilon application in diagnosis and therapy of myasthenia gravis. J Neurol.

[10] Castagna A., Ferrara L., Asnaghi E., Rega V., Fiorini G. (2019). Functional limitations, and cognitive impairment predict the outcome of dysphagia in older patients after an acute neurologic event. Neuro Rehabil.

[11] Tangalos E.G., Petersen R.C. (2018). Mild cognitive impairment in geriatrics. Clin Geriatr Med.

[12] Penner I.K., Paul F. (2017). Fatigue as a symptom or comorbidity of neurological diseases. Nat Rev Neurol.

[13] Siciliano M., Trojano L., Santangelo G., De Micco R., Tedeschi G., Tessitore A. (2018). Fatigue in Parkinson’s disease: a systematic review and meta-analysis. Mov Disord.

[14] Farrugia M.E., Di Marco M., Kersel D., Carmichael C. (2018). A physical and psychological approach to managing fatigue in myasthenia gravis: a pilot study. J Neuromuscul Dis.

[15] Aviv J.E., Kim T., Sacco R.L., Kaplan S., Goodhart K., Diamond B. (1998). FEESST: a new bedside endoscopic test of the motor and sensory components of swallowing. Ann Otol Rhinol Laryngol.

[16] Kamarunas E.E., McCullough G.H., Guidry T.J., Mennemeier M., Schluterman K. (2014). Effects of topical nasal anesthetic on fiberoptic endoscopic examination of swallowing with sensory testing (FEESST). Dysphagia.

[17] Costa M.M. (2010). Videofluoroscopy: the gold standard exam for studying swallowing and its dysfunction. Arq Gastroenterol.

[18] The Speech Pathology Association of Australia Limited (2013).

[19] Madden C., Fenton J., Hughes J., Timon C. (2000). Comparison between videofluoroscopy and milk-swallow endoscopy in the assessment of swallowing function. Clin Otolaryngol Allied Sci.

[20] Cuschieri S. (2019). The STROBE guidelines. Saudi J Anaesth.

[21] Mandysova P., Skvrňáková J., Ehler E., Cerný M. (2011). Development of the brief bedside dysphagia screening test in the Czech Republic. Nurs Health Sci.

[22] Leder S.B., Suiter D.M., Agogo G.O., Cooney L.M. (2016). An epidemiologic study on ageing and dysphagia in the acute care geriatric-hospitalized population: a replication and continuation study. Dysphagia.

[23] de Lima Alvarenga E.H., Dall’Oglio G.P., Murano E.Z., Abrahão M. (2018). Continuum theory: presbyphagia to dysphagia? Functional assessment of swallowing in the elderly. Eur Arch Otorhinolaryngol.

[24] Braun T., Juenemann M., Viard M., Meyer M., Reuter I., Prosiegel M. (2019). Adjustment of oral diet based on flexible endoscopic evaluation of swallowing (FEES) in acute stroke patients: a cross-sectional hospital-based registry study. BMC Neurol.

[25] Suntrup-Krueger S., Schmidt S., Warnecke T., Steidl C., Muhle P., Schroeder J.B. (2019). Extubation readiness in critically ill stroke patients. Stroke.

[26] Warnecke T., Oelenberg S., Teismann I., Hamacher C., Lohmann H., Ringelstein E.B. (2010). Endoscopic characteristics and levodopa responsiveness of swallowing function in progressive supranuclear palsy. Mov Disord.

[27] Nienstedt J.C., Buhmann C., Bihler M., Niessen A., Plaetke R., Gerloff C. (2018). Drooling is no early sign of dysphagia in Parkinson’s disease. Neurogastroenterol Motil.

[28] Pflug C., Bihler M., Emich K., Niessen A., Nienstedt J.C., Flügel T. (2018). Critical dysphagia is common in Parkinson disease and occurs even in early stages: a prospective cohort study. Dysphagia.

[29] Warnecke T., Ritter M.A., Kroger B., Oelenberg S., Teismann I., Heuschmann P.U. (2009). Fiberoptic endoscopic Dysphagia severity scale predicts outcome after acute stroke. Cerebrovasc Dis.

[30] Shapira-Galitz Y., Yousovich R., Halperin D., Wolf M., Lahav Y., Drendel M. (2019). Does the Hebrew Eating Assessment Tool-10 correlate with pharyngeal residue, penetration and aspiration on fiberoptic endoscopic examination of swallowing?. Dysphagia.

[31] D’Ottaviano F.G., Linhares Filho T.A., Andrade H.M., Alves P.C., Rocha M.S. (2013). Fiberoptic endoscopy evaluation of swallowing in patients with amyotrophic lateral sclerosis. Braz J Otorhinolaryngol.

[32] Marian T., Schröder J., Muhle P., Claus I., Oelenberg S., Hamacher C. (2017). Measurement of oxygen desaturation is not useful for the detection of aspiration in dysphagic stroke patients. Cerebrovasc Dis Extra.

[33] Gozzer M.M., Cola P.C., Onofri S.M.M., Merola B.N., Silva R.G.D. (2019). Fiberoptic endoscopic findings of oropharyngeal swallowing of different food consistencies in Amyotrophic Lateral Sclerosis. Achados videoendoscópicos da deglutição em diferentes consistências de alimento na Esclerose Lateral Amiotrófica. CoDAS.

[34] Imaizumi M., Suzuki T., Matsuzuka T., Murono S., Omori K. (2019). Low-risk assessment of swallowing impairment using flexible endoscopy without food or liquid. Laryngoscope.

[35] Schröder J.B., Marian T., Claus I., Muhle P., Pawlowski M., Wiendl H. (2019). Substance P saliva reduction predicts pharyngeal dysphagia in parkinson’s disease. Front Neurol.

[36] Pilz W., Baijens L.W., Passos V.L., Verdonschot R., Wesseling F., Roodenburg N. (2014). Swallowing assessment in myotonic dystrophy type 1 using fiberoptic endoscopic evaluation of swallowing (FEES). Neuromuscul Disord.

[37] Somasundaram S., Henke C., Neumann-Haefelin T., Isenmann S., Hattigen E., Lorenz M.W. (2014). Dysphagia risk assessment in acute left-hemispheric middle cerebral artery stroke. Cerebrovasc Dis.

[38] Souza G.A.D. de, Gozzer M.M., Cola P.C., Onofri S.M.M., Gonçalves da Silva R. (2019). Desempenho longitudinal da deglutição orofaríngea na distrofia miotônica tipo 1. Audiol Commun Res.

[39] Souza G.A.D. de, Silva R.G. da, Cola P.C., Onofri Suely M.M. (2019). Resíduos faríngeos nas disfagias orofaríngeas neurogênicas. CoDAS.

[40] Umay E.K., Karaahmet F., Gurcay E., Balli F., Ozturk E., Karaahmet O. (2018). Dysphagia in myasthenia gravis: the tip of the Iceberg. Acta Neurol Belg.

[41] Farneti D., Fattori B., Bastiani L. (2019). Time as a factor during endoscopic assessment of swallowing: relevance in defining the score and severity of swallowing disorders. Acta Otorhinolaryngol Ital.

[42] Dziewas R., Warnecke T., Olenberg S., Teismann I., Zimmermann J., Kramer C. (2008). Towards a basic endoscopic assessment of swallowing in acute stroke — development and evaluation of a simple dysphagia score. Cerebrovasc Dis.

[43] Langmore S.E., Terpenning M.S., Schork A., Chen Y., Murray J.T., Lopatin D. (1998). Predictors of aspiration pneumonia: how important is dysphagia?. Dysphagia.

[44] Hyodo M., Nishikubo K., Hirose K. (2010). New scoring proposed for endoscopic swallowing evaluation and clinical significance [in Japanese]. Nihon Jibiinkoka Gakkai Kaiho.

[45] ADA: American Dietetic Association (2002).

[46] Prosiegel M., Heintze M., Wagner-Sonntag E., Hannig C., Wuttge-Hannig A., Yassouridis A. (2002). Deglutition disorders in neurological patients. A prospective study of diagnosis, pattern of impairment, therapy and outcome. Der Nervenarzt.

[47] Clark H.M., Stierwalt J.A.G., Tosakulwong N., Botha H., Ali F., Whitwell J.L. (2020). Dysphagia in progressive supranuclear palsy [published online ahead of print, 2019 Nov 1]. Dysphagia.

[48] Jani M.P., Gore G.B. (2016). Swallowing characteristics in Amyotrophic Lateral Sclerosis. Neuro Rehabilitation.

[49] Lee W.K., Yeom J., Lee W.H., Seo H.G., Oh B.M., Han T.R. (2016). Characteristics of dysphagia in severe traumatic brain injury patients: a comparison with stroke patients. Ann Rehabil Med.

